# Pharmacogenomics of Major Depressive Disorder in Indigenous Amazonian Populations

**DOI:** 10.1002/cpt.70207

**Published:** 2026-01-16

**Authors:** Kaio Evandro Cardoso Aguiar, Natasha Monte da Silva, Juliana Carla Gomes Rodrigues, André Maurício Ribeiro‐dos‐Santos, Sandro José de Souza, Ândrea Ribeiro‐dos‐Santos, João Farias Guerreiro, Sidney Emanuel Batista dos Santos, Marianne Rodrigues Fernandes, Ney Pereira Carneiro dos Santos

**Affiliations:** ^1^ Oncology Research Center Federal University of Pará Belém, Pará PA Brazil; ^2^ Laboratory of Human and Medical Genetics, Institute of Biological Science Federal University of Pará Belém, Pará Brazil; ^3^ Brain Institute Federal University of Rio Grande do Norte Natal RN Brazil

## Abstract

Major depressive disorder is a highly prevalent psychological disorder worldwide and its main treatment is the use of Selective Serotonin Reuptake Inhibitors. However, few studies have demonstrated the relationship between the presence of genetic variants in pharmacogenes and the efficacy of these drugs, especially in populations with a unique genetic profile, such as the Indigenous peoples of the Amazon. Our study characterized the molecular profile of nine genes related to drug administration, metabolization, distribution, and elimination pathways and pharmacodynamic mechanisms of drug response through Whole Exome Sequencing applied in 64 Indigenous located in the Amazon. We compared the allele frequencies of the variants in Indigenous peoples and other world populations using Fisher's exact test carried out in RStudio v.3.5.1. We identified a total of 125 variants, of which 6 are possible new variants in our population on the *HTR2A*, *HTR2C*, *CYP2D6*, and *CYP1A2* genes. At least 9 variants showed a significant difference in the Indigenous population compared with other populations worldwide. Our study reaffirms the unique genetic profile of the Brazilian Amazon Indigenous population and allows us to contribute population‐specific variants that may serve as future pharmacogenomic biomarkers that help in the understanding of the individual genetic profiles of Indigenous people. Although the present study does not evaluate clinical drug response, the characterization of these variants provides a foundation for future studies exploring their potential impact on antidepressant efficacy in Indigenous populations and the application of this knowledge in the development of specific treatment protocols guided by pharmacogenomics.

**Study Highlights**

**WHAT IS THE CURRENT KNOWLEDGE ON THE TOPIC?**

The knowledge about genetic variants and their impact on drug effectiveness is mainly focused on populations of European origin, and the protocols developed are often inadequate when aimed at genetically distinct populations, such as Indigenous Amazonians. Large‐scale studies using genome‐wide analysis (GWAS) and next‐generation sequencing (NGS) are rare in these populations, limiting understanding of diseases and therapeutic efficacy, especially in the context of genomic medicine.

**WHAT QUESTION DID THE STUDY ADDRESS?**

This study investigated the presence of genetic variants in 9 key genes in routes of administration, distribution, metabolism, and elimination of drugs for the treatment of major depressive disorder in Indigenous Amazonian populations with a unique genetic profile.

**WHAT DOES THE STUDY ADD TO OUR KNOWLEDGE?**

This research identified genetic variants of potential pharmacogenomic relevance in Indigenous populations, including possible new variants that may be specific to this group. Although our study does not evaluate pharmacological efficacy, these findings highlight variants that justify the importance of future functional and clinical investigations to determine their potential impact on response to antidepressants in populations with a different genetic profile.

**HOW MIGHT THIS CHANGE CLINICAL PHARMACOLOGY OR TRANSLATIONAL SCIENCE?**

Knowledge of these genetic variants and their implications can enable the implementation of public policies that promote precision health for Indigenous people and must begin with an in‐depth study of their variability. It is essential to investigate molecular biomarkers specific to these populations, with their practical application moving into the clinic.

Major depressive disorder (MDD) is characterized by a feeling of melancholy or sadness severe enough to affect daily tasks.^1^ Depression, responsible for almost 29% of mental disorders globally, is the leading psychiatric disorder contributing to years lived with functional disability among adults. Its high potential to cause disability places it in this position, as documented in the largest review on mental health conducted by the World Health Organization (WHO).^2^

Selective serotonin reuptake inhibitors (SSRIs) are the first treatment choices for depression, including citalopram, escitalopram, fluoxetine, and others. Although these drugs have the same mechanism of action, several variables can lead to adverse effects and impact their effectiveness.^3^

Pharmacogenomic (PGx) research into genetic variants in key genes of the Administration, Distribution, Metabolism, and Elimination (ADME) pathways of drugs can explain why drugs have low or zero efficacy and side effects in certain individuals [Bachtiar]. Genetic single nucleotide variants (SNVs) in genes encoding cytochrome P450 (CYP) enzymes have previously been reported in studies on ADME pathways as directly relevant to antidepressant drug degradation and efficacy.^4^ The CYP family is responsible for metabolizing about 75% of the drugs, including the vast majority of psychotropic drugs. Genetic variations in these genes can lead to different degrees of drug metabolism in a patient, from a “normal metabolizer” to a “poor metabolizer” or “ultra‐rapid metabolizer.” Such differences directly impact the concentration of the drug: A poor metabolizer may accumulate the active compound or its precursor to potentially toxic levels, whereas an ultra‐rapid metabolizer may eliminate the drug so quickly that therapeutic efficacy is lost.^5^, ^6^ This is particularly critical for prodrugs, whose activation depends on specific metabolic pathways; in such cases, insufficient enzyme activity can prevent drug activation, while excessive activity can lead to supratherapeutic concentrations of the active metabolite.^7^ The development of PGx in psychiatry indicates the effectiveness of preventive genotyping for more successful psychotherapy.^8^

Another factor contributing to this scenario is the ancestral genetic influence of different genetic backgrounds in the formation of a population, having a major impact on pharmacogenomic research.^9^ Studies show that variability in populations directly affects the allele frequency of pharmacogenomic variants that can influence specific responses to drug efficacy, dosage, and toxicity.^10^, ^11^

The historical formation of the Brazilian population is one of the most heterogeneous in the world, with genetic contributions coming from different continents and, above all, from those who were already here, the Indigenous people. Specifically in the north of Brazil, the ancestry of these original peoples significantly contributes to the regional population's genetic makeup, influencing the distribution of pharmacogenomic variants relevant to drug response and giving rise to a population with a unique genetic profile.^12^ The influence of Indigenous genetics has already been recognized in various clinical studies as exerting different responses to standard treatments for various diseases.^13^, ^14^

Despite growing data on depression around the world and the apparent influence of genetics on various treatments, little pharmacogenomic data are focused on traditional Indigenous populations. In this way, the identification of genetic variants that are important for clinical practice in these populations will be essential for the development of effective treatment protocols not only for this group but also for the entire mixed Brazilian population.^15^

Research papers in neuropsychopharmacology using Genome‐Wide Association Studies (GWAS) have identified different genes that are important for clinical treatment and dosage in psychiatric disorders. These include the *HTR2A* and *HTR2C* genes, which encode serotonin receptors; the *SLC1A1* gene, which encodes an important protein in glutamate reuptake in the synapses of the central nervous system; the *SLC6A4* gene, which encodes serotonin reuptake transporter enzymes; the *COMT* gene, which encodes drug metabolizing enzymes and an important transporter enzyme; and the *ABCB1* gene, which controls drug uptake in the brain.^8^, ^16^

In this context, our study initiates research into neuropsychopharmacology in Indigenous Amazonian populations, becoming a pioneer in this area among populations that are difficult to access. We sought to characterize and investigate genetic variants in the whole exome of nine key genes related to antidepressant pharmacogenomics: four genes primarily involved in the absorption, distribution, metabolism, and elimination (ADME)—*ABCB1, CYP1A2, CYP2B6*, and *CYP2D6*—and five genes related to pharmacodynamic (PD) mechanisms of drug response—*COMT, HTR2A, HTR2C, SLC1A1*, and *SLC6A4*, in pathways of drugs in the treatment of major depressive disorder (MDD), in order to explore potential genetic factors that could contribute to interindividual differences in therapeutic response. The identification of population‐specific pharmacogenomic variants may help to explain possible differences in treatment outcomes observed in Indigenous populations in the Brazilian Amazon.

## METHODS

### Population analysis for the study

The study consisted of samples from a population of 64 Indigenous Amazonians from the northern region of Brazil, represented by 12 different Indigenous peoples: Asurini located in the Xingu and Tocantins, Arara, Araweté, Awa‐Guajá, Juruna, Kayapó/Xikrin, Karipuna, Munduruku, Phurere, Wajãpi, and Zo'é. For the statistical analyses, all 64 Indigenous people were grouped into a single group called (INDG). Genetic ancestry data were obtained from a panel of 64 Ancestry Informative Markers (IAM), as described in Ramos *et al*.^17^ All participants and their village leaders were instructed about the research to be carried out, as well as signing an Informed Consent Form (ICF). The participation was strictly voluntary; the researchers held project presentation meetings with the participants and their respective leaders. During these meetings, the objective, methodology, risks, and benefits of the research were explained clearly and accessibly. Furthermore, explicitly detailing access routes, leaders' names, or precise logistical contact methods for each of the 12 different Indigenous ethnic groups could compromise the privacy and security of these communities, exposing information that could facilitate unauthorized contact or exploitation by third parties. Discretion regarding field logistics is a protective measure adopted in research with isolated or hard‐to‐reach populations in the Amazon.

The frequency of Indigenous populations was compared with other continental populations (Europe (EUR), Africa (AFR), East Asia (EAS), South Asia (SAS), and the Americas (AMR)) available in the 1,000 Genomes Database, version 3 (available at: http://www.1000genomes.org; accessed on May 10, 2024). The study included 503 individuals from Europe, 661 from Africa, 504 from East Asia, 489 from South Asia, and 347 from the Americas.

### DNA extraction and exome analysis

DNA extraction was carried out according to the phenol‐chloroform method^18^ with the appropriate modifications. Quantification of the extraction products was carried out using a Nanodrop‐8,000 spectrophotometer (Thermo Fisher Scientific Inc., Wilmington, DE, USA), and subsequent analysis to filter the quality of the extracted material was carried out using 2% agarose gel electrophoresis.

The variant library (exome) was prepared using Nextera Rapid Capture Exome (Illumina®, San Diego, CA, USA) and SureSelect Human All Exon V6 (Agilent Technologies, Santa Clara, CA, USA), following the kit protocol provided by the manufacturer. The sequencing reaction was performed by the NextSeq 500® platform (Illumina®, San Diego, CA, USA) using the NextSeq 500 High‐output v2 Kit 300 cycle kit (Illumina®, San Diego, CA, USA).

### Selection of genes

The genes were selected by consulting the PubMed database (pubmed.ncbi.nlm.nih.gov; accessed January 20, 2024) and the PharmGKB database (pharmgkb.org; accessed January 20, 2023). The nine genes selected, four genes primarily involved in pharmacokinetic (ADME) processes—*ABCB1*, *CYP1A2*, *CYP2B6*, and *CYP2D6*—and five genes related to pharmacodynamic (PD) mechanisms of drug response—*COMT*, *HTR2A*, *HTR2C*, *SLC1A1*, and *SLC6A4*, are among the most cited in the literature and psychotropic drug pathways. The function of these genes, as well as the associated drugs, is summarized in **Table** **1**.

**Table 1:** Descriptive summary of the 9 selected genes and related psychiatric drugs

Gene	Description	Associated drug
*ABCB1*	This gene encodes P‐glycoprotein (P‐gp), a transport protein located in the blood–brain barrier. P‐gp “pumps” drugs out of the brain. Variants in the *ABCB1* gene can affect P‐gp's ability to transport drugs, altering their concentration in the brain. Studies show that specific *ABCB1* genotypes are associated with therapeutic response and side effects of antidepressants, indicating that patients with lower P‐gp activity may have higher concentrations of the drug in the brain, with a greater risk of side effect	Sertaline, Paroxetine, Velanfaxine, Escitalopram^19^, ^24^
*COMT*	This gene, which has been extensively studied in psychiatric disorders, catalyzes the O‐methylation of catecholamines such as dopamine. Several genetic variants in this gene have been linked to psychiatric disorders and pharmacological response	Olanzapine, Clozapine, Risperidone, Metilfenidate^20^, ^21^
*CYP1A2*	Cytochrome P450 enzymes metabolize various psychiatric drugs, including some antidepressants and antipsychotics. The activity of this enzyme can vary greatly between individuals, and polymorphisms, such as ‐163C>A, can influence the efficacy and safety of these drugs.	Clozapine, Olanzapine, Duloxetine.^22^, ^23^
*CYP2B6*	This highly polymorphic gene with 35 alleles starred by PharmVar and with significantly different allele frequencies between populations is categorized as normal function (e.g., CYP2B6*1), decreased function (e.g., CYP2B6*6 and *9), no function (e.g., CYP2B6*18), and increased function (e.g., CYP2B6*4)	Bupropiona, Sertralina (to a lesser extent)^5^, ^23^, ^24^
*CYP2D6*	This gene, which significantly influences the pharmacokinetics or efficacy of relevant psychiatric drugs, encodes important metabolizing enzymes. Genetic variants in this gene are divided into normal metabolizers (NMs) carry gene variants which encode “normal” CYP2C19 or *CYP2D6* enzyme capacity; poor metabolizers (PMs) lack the functional CYP gene in question, having no enzyme activity; intermediate metabolizers (IMs) carry partially defective combinations of CYP alleles and have reduced enzyme activity; and ultra‐rapid metabolizers (UMs) exhibit higher‐than‐normal metabolic capacity, sometimes due to gene duplications	Paroxetina, Fluoxetina, Fluvoxamina, Venlafaxina, Vortioxetina^23^, ^24^
*HTR2A*	This gene encodes a postsynaptic serotonin receptor–2A; genetic variants have been associated with response to treatment with antidepressants. Some studies indicate that certain genotypes can predict the effectiveness of drugs such as selective serotonin reuptake inhibitors (SSRIs)	Citalopram, Escitalopram, Fluoxetina, Sertralina^24^
*HTR2C*	This gene encodes a postsynaptic serotonin receptor–2C. Studies show its association with the risk of metabolic disorders in patients using clozapine	Clozapina, Olanzapina.^25^
*SLC1A1*	This gene, which encodes an important glutamate transporter, has been linked in studies to psychiatric disorders and side effects in antipsychotic drug treatments. Studies have mainly identified its association with obsessive‐compulsive disorder (OCD), but new approaches relate this gene to depression, via the role of glutamate in its pathophysiology	Fluvoxamina, Sertralina^26^, ^27^
*SLC6A4*	This gene encodes a serotonin reuptake transporter (5‐HTT) that acts by reuptaking the neurotransmitter from synaptic spaces into presynaptic neurons. SSRIs bind directly to the transporter, blocking serotonin reuptake in the gene promoter region. Long allele variations have been associated with serotonin reuptake activity that is 1.9 to 2.2 times higher than that of the short allele	Sertralina, Paroxetina, Escitalopram, Vortioxetina, Vilazodona^24^

### Selection of variants

The selection of variants resulted from the use of two evaluation criteria. Firstly, a minimum of 10 coverage readings was required for each variant investigated. Secondly, the categorization of variants considering only those with a high risk, moderate, or modifier effect, according to the SNPeff classification (https://pcingola.github.io/SnpEff/; accessed May 5, 2024). The SNPeff software predicts functional consequences based on the type and genomic position of each variant. This includes the assessment of coding disruptions, amino acid changes, splicing site alterations, and regulatory region variants, allowing for a biologically informed estimate of functional impact. As a result of the exome analysis, 125 variants were found, as described in **Table** **S1**. After selection based on the aforementioned criteria, a total of 101 variants remained to be followed in the study's investigation process.

### Statistical and bioinformatics analysis

The study population's allele frequency was obtained by calculating the genes investigated and compared with other populations (EUR, AMR, EAS, SAS, and AFR). Fisher's exact test was used to assess the statistical significance of the different frequencies between populations, and Hochberg's test was used to correct for multiple comparisons. The population variability of polymorphisms was corrected by the Wright fixation index (FST). A *P*‐value of ≤0.05 was considered significant for the results. All research was carried out in RStudio v.3.5.1.

Bioinformatic analyses evaluated the quality and filtering of FASTQ reads, alignment with the GRCh38 reference genome, post‐alignment processing (including duplicate removal, base quality recalibration, and local realignment), variant identification using GATK, and subsequent functional annotation added to the ViVa® software, following a standardized workflow previously applied in studies of Amazonian populations described by Cohen‐Paes *et al*.^28^

### Ethics statement

The study was approved by the National Research Ethics Committee (CONEP) and by the Research Ethics Committee of the Center for Tropical Medicine at the Federal University of Pará (CAE: 20654313.6.0000.5172).

## RESULTS

**Figure 1:**
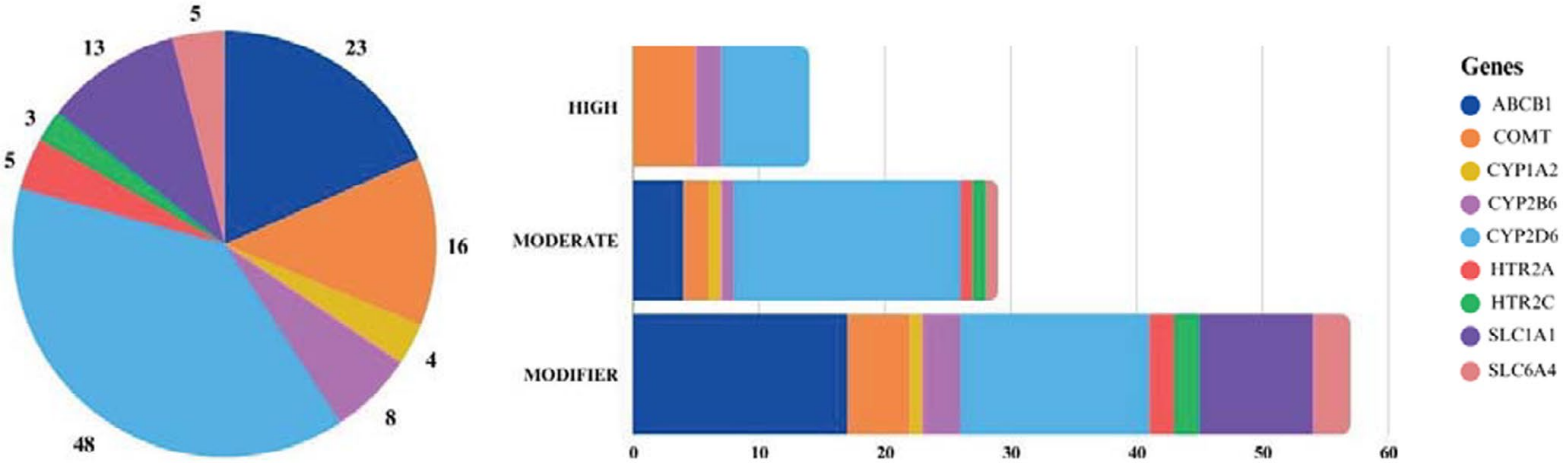
Raw data on the contribution of the genetic variants analyzed in each gene and predicted impact.

After investigating the 9 genes, 101 genetic variants went on to be analyzed following the evaluation criteria, of which 14 are of high impact, 30 of moderate impact, and 57 of modifying impact. Of these, 23 belong to the *ABCB1* gene, 16 to the *COMT* gene, 4 to the *CYP1A2* gene, 8 to the *CYP2B6* gene, 48 to the *CYP2D6* gene, 5 to the *HTR2A* gene, 3 to the *HTR2C* gene, 13 to the *SLC1A1* gene, and 5 to the *SLC6A4* gene (**Figure** **1**).

**Table** **2** describes the general characteristics of the high and moderate impact variants predicted by the SNPeff software, including the affected gene, chromosome, chromosomal position, consequence, the type of variant, reference ID, clinical impact, and the frequency of the smallest allele for the Indigenous population and the five large populations described by the 1,000 genomes database (AFR, AMR, EAS, EUR, and SAS). The other variants with modifying impact and low impact, as well as their characteristics, are described in **Table** **S1**.

**Table 2:** Descriptions of the variants in the *ABCB1*, *COMT*, *CYP1A2*, *CYP2B6*, *CYP2D6*, *HTR2A*, *HTR2C*, *SLC1A1,* and *SLC6A4* genes according to high impact and moderate, in addition to continental populations (African (AFR), American population (AMR), East Asian (EAS), European (EUR), and South Asian (SAS)) described in the 1,000 genomes database

Gene	Chromosome	Position	Reference	Variant	Var Type	SNP Id	Impact	INDG*	AFR*	AMR*	EAS*	EUR*	SAS*
*ABCB1*	Chr7	87,509,343	A	T	Snv	rs2229107	Moderate	0.0	0.072	0.006	**—**	**—**	**—**
*ABCB1*	Chr7	87,531,302	C	A	Snv	rs2032582	Moderate	0.0	0.020	0.369	0.398	0.410	0.592
*ABCB1*	Chr7	87,504,335	C	T	Snv	rs28364274 rs45456698	Moderate	0.0	**—**	0.046	0.003	**—**	**—**
*ABCB1*	Chr7	87,531,302	A	C	Snv	rs2032582	Moderate	0.0	0.020	0.369	0.398	0.410	0.592
*COMT*	Chr22	19,963,684	C	G	Snv	rs4818	High	0.023	0.170	0.295	0.341	0.403	0.315
*COMT*	Chr22	19,962,740	G	T	Snv	rs6267	High	0.116	0.001	0.033	0.035	0.007	0.001
*COMT*	Chr22	19,962,745	G	A	Snv	rs740602	High	0.008	0.200	0.020	**—**	0.009	0.012
*COMT*	Chr22	19,962,712	C	T	Snv	rs4633	High	0.453	0.293	0.380	0.270	0.499	0.445
*COMT*	Chr22	19,963,748	G	A	Snv	rs4680	High	0.453	0.281	0.378	0.280	0.500	0.441
*COMT*	Chr22	19,962,740	G	T	Snv	rs6267	Moderate	0.116	0.001	0.033	0.035	0.007	0.001
*COMT*	Chr22	19,963,748	G	A	Snv	rs4680	Moderate	0.453	0.281	0.378	0.280	0.500	0.441
*CYP1A2*	Chr15	74,751,251	C	A	Snv	rs17861157	Moderate	0.083	0.089	0.003	**—**	**—**	**—**
*CYP2B6*	Chr19	41,006,936	G	T	Snv	rs3745274	High	0.0	0.374	0.373	0.215	0.236	0.381
*CYP2B6*	Chr19	41,012,413	C	T	Snv	rs140830969	High	0.083	‐	0.004	**—**	**—**	**—**
*CYP2B6*	Chr19	41,006,936	G	T	Snv	rs3745274	Moderate	0.0	0.374	0.373	0.215	0.236	0.381
*CYP2D6*	Chr22	42,127,458	G	A	Snv	rs146271511	High	0.083	**—**	**—**	**—**	**—**	**—**
*CYP2D6*	Chr22	42,142,513	T	TC	Indel	rs149012039	High	0.101	0.489	0.231	0.571	0.297	0.274
*CYP2D6*	Chr22	42,127,556	T	C	Snv	rs202102799	High	0.0833	**—**	**—**	**—**	**—**	**—**
*CYP2D6*	Chr22	42,127,526	C	T	Snv	rs1058172	High	0.083	**—**	**—**	**—**	**—**	**—**
*CYP2D6*	Chr22	42,130,692	G	A	Snv	rs1065852	High	0.083	0.113	0.148	0.571	0.202	0.165
*CYP2D6*	Chr22	42,127,537	A	G	Snv	rs28371726	High	0.083	**—**	**—**	**—**	**—**	**—**
*CYP2D6*	Chr22	42,128,945	C	T	Snv	rs3892097	High	0.028	0.0061	0.130	0.002	0.186	0.109
*CYP2D6*	Chr22	42,127,512	C	T	Snv	rs61745683	Moderate	0.083	**—**	**—**	**—**	**—**	**—**
*CYP2D6*	Chr22	42,144,356	G	T	Snv	rs116100811	Moderate	0.083	0.116	0.003	**—**	**—**	**—**
*CYP2D6*	Chr22	42,128,173	CCTT	C	Indel	rs5030656rs869035800	Moderate	0.0	0.001	0.013	**—**	0.026	**—**
*CYP2D6*	Chr22	42,127,608	C	T	Snv	rs59421388	Moderate	0.083	0.107	0.003	**—**	**—**	**—**
*CYP2D6*	Chr22	42,142,507	A	G	Snv	rs3021082	Moderate	0.166	0.489	0.228	0.569	0.294	0.274
*CYP2D6*	Chr22	42,127,526	C	T	Snv	rs1058172	Moderate	0.083	**—**	**—**	**—**	**—**	**—**
*CYP2D6*	Chr22	42,127,503	C	T	Snv	rs61737946	Moderate	0.083	**—**	**—**	**—**	**—**	**—**
*CYP2D6*	Chr22	42,140,331	C	T	Snv	rs3171709	Moderate	0.016	0.105	0.003	**—**	**—**	**—**
*CYP2D6*	Chr22	42,127,556	T	C	Snv	rs202102799	Moderate	0.083	**—**	**—**	**—**	**—**	**—**
*CYP2D6*	Chr22	42,141,636	G	C	Snv	rs113472173	Moderate	0.083	0.212	0.009	**—**	0.002	**—**
*CYP2D6*	Chr22	42,140,302	G	A	Snv	rs2743456	Moderate	0.083	0.107	0.144	0.548	0.201	0.158
*CYP2D6*	Chr22	42,141,633	G	A	Snv	rs79164577	Moderate	0.083	0.213	0.009	**—**	0.002	0.001
*CYP2D6*	Chr22	42,129,132	C	T	Snv	rs61736512	Moderate	0.083	0.110	0.003	**—**	**—**	**—**
*CYP2D6*	Chr22	42,130,692	G	A	Snv	rs1065852	Moderate	0.083	0.113	0.148	0.571	0.202	0.165
*CYP2D6*	Chr22	42,127,458	G	A	Snv	rs146271511	Moderate	0.083	**—**	**—**	**—**	**—**	**—**
*CYP2D6*	Chr22	42,129,770	G	A	Snv	rs28371706	Moderate	0.083	0.218	0.009	**—**	0.002	**—**
*CYP2D6*	Chr22	42,140,642	T	C	Snv	rs2070907	Moderate	0.083	**—**	**—**	**—**	**—**	**—**
*CYP2D6*	Chr22	42,141,270	T	C	Snv	rs28367005	Moderate	0.083	0.011	0.052	0.019	0.112	0.151
*HTR2A*	Chr13	46,834,899	G	A	Snv	rs6314	Moderate	0.015	0.121	0.079	0.005	0.080	0.076
*HTR2C*	ChrX	114,731,382	G	C	Snv	rs143566182	Moderate	0.0	0.003	**—**	**—**	**—**	**—**
*SLC6A4*	Chr17	30,221,792	C	G	Snv	rs6355	Moderate	0.083	**—**	0.004	**—**	0.018	0.014

*, minor allele frequencies; −, no annotation; AFR, African population; AMR, American population; EAS, East Asian population; EUR, European population; INDG, indigenous population; SAS, South Asian population.

Of the variants shown in **Table** **2**, 14 had a high predicted impact (rs4818, rs6267, rs740602, rs4633, rs4680, rs3745274, rs140830969, rs146271511, rs149012039, rs202102799, rs1058172, rs1065852, rs28371726, and rs3892097) present in the *COMT*, *CYP2B6*, and *CYP2D6* genes. The high‐impact rs149012039 in the *CYP2D6* gene at position 42,142,513 is characterized by an insertion/deletion causing a change in the reading matrix and has a high frequency in the Indigenous population, over 10% of individuals. Other noteworthy variants are rs4633 and rs4680, both in the *COMT* gene, characterized by being SNVs with a frequency of over 45% in the study population. In addition, another 6 new variants possibly exclusive to the Indigenous Amazonian population were reported in the investigation, present in the *HTR2A*, *HTR2C*, *CYP2D6*, and *CYP1A2* genes (**Table** **3**).

**Table 3:** Description of new variants found in the Indigenous population to genes relevant to drug pathways for depression

Gene	Chromosome^#^	Position	Var type	Region detailed	Reference	Variant	Impact	Change protein	MAF*
*HTR2A*	Chr13	46,896,758	Indel	Utr 5 Prime	TCC	T	Modifier	c.‐415_‐414delGG	0.100
*HTR2C*	ChrX	114,731,282	Snv	Intron	C	A	Modifier	c.36‐12C>A	0.200
*CYP2D6*	Chr22	52,919	Snv	Intron	G	C	Modifier	c.180 + 34C>G	0.661
*CYP2D6*	Chr22	52,912	Snv	Intron	T	G	Modifier	c.180 + 41A>C	0.616
*CYP2D6*	Chr22	52,910	Snv	Intron	C	G	Modifier	c.180 + 43G>C	0.530
*CYP1A2*	Chr15	74,754,842	Snv	Synonymous Coding	C	A	Low	p.Ala435Ala	0.017

(#) Reference genome to chromosomal location obtained from the GH38 of the human genome from the Human Genome Project; (*) minor allele frequency in Indigenous population.

Of the new variants found in Indigenous populations, five have a modifying impact and one has a low impact. The 3 variants in the *CYP2D6* gene have a high allelic frequency (MAF > 0.5) in the Indigenous population, present in intronic regions, as does the variant found in the *HTR2C* gene at position 114,731,282 (MAF = 0.200). In addition, the variant at position 46,896,758 (MAF = 0.100) found in the *HTR2A* gene, present in a 5′UTR region, was also observed. All five impact modifier variants are present in regions that aid gene expression and regulation.

**Figure 2:**
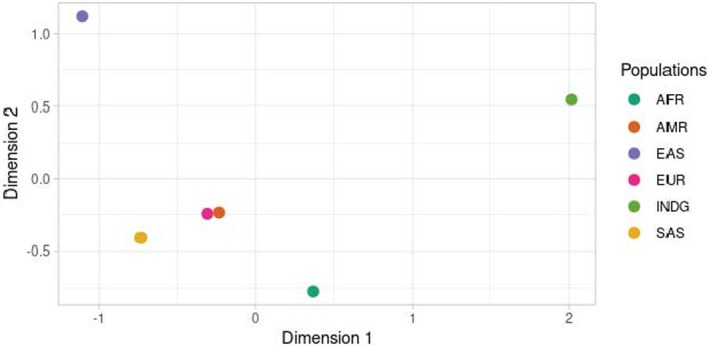
Multidimensional scale analysis (MDS) plot illustrating Indigenous population (INDG) and continental population (African (AFR), American (AMR), East Asian (EAS), European (EUR), and South Asian (SAS)) according to genetic traits of variants in the investigated genes.

We used the Multidimensional Scale Analysis (MDS) graph using FST values and the allele frequency of the genetic variants in the populations to compare the 101 variants found in the genes and illustrate the distance between them (**Figure** **2**).

It is possible to observe that the INDG population appears in a field isolated from the others, demonstrating the unique genetic profile of this group to the other populations based on the variants investigated. The populations genetically closest to the INDG in this exome were the AFR, followed by the AMR population, made up mainly of Latin American individuals. The EAS group was the most distant among the populations investigated.

**Table** **4** shows paired comparisons between the frequencies observed in the INDG population and the other global populations, using Fisher's exact test. All the inserted variants have a high or moderate impact, as well as significant differences (*P*‐value ≤ 0.05) in at least three of the five populations. The comparative analysis of the genetic variants with a modifying impact is shown in **Table** **S2**.

**Table 4:** Paired comparison (*P*‐value) with significant results of allele frequencies of genetic variants with high and moderate impact in the Indigenous population (INDG) and continental populations (African (AFR), American (AMR), East Asian (EAS), European (EUR), and South Asian (SAS)) described in the 1,000 genomes database

Gene	SNP Id	Var type	Impact	INDG vs. AFR*	INDG vs. AMR*	INDG vs. EAS*	INDG vs. EUR*	INDG vs. SAS*
*ABCB1*	rs2032582	Snv	Moderate	—	**5.63e‐04**	**2.42e‐05**	**1.08e‐05**	**1.80e‐15**
*COMT*	rs4818	Snv	High	**2.29e‐04**	**3.31e‐09**	**3.02e‐11**	**3.15e‐14**	**7.09e‐10**
*COMT*	rs6267	Snv	High	**1.25e‐04**	**2.73e‐02**	**2.73e‐02**	**9.89e‐04**	**1.25e‐04**
*CYP2B6*	rs3745274	Snv	High	**3.10e‐16**	**3.10e‐16**	**6.95e‐09**	**1.43e‐09**	**1.40e‐16**
*CYP2D6*	rs149012039	Indel	High	**3.11e‐05**	**1.36e‐02**	**5.57e‐09**	**4.11e‐04**	**1.80e‐03**
*CYP2D6*	rs28371706	Snv	Moderate	**1.08e‐02**	**1.09e‐02**	—	**1.88e‐03**	—
*CYP2D6*	rs3021082	Snv	Moderate	**1.33e‐07**	—	**9.17e‐11**	**3.02e‐02**	—
*CYP2D6*	rs3892097	Snv	High	—	**1.08e‐02**	—	**2.37e‐04**	**4.25e‐02**
*CYP2D6*	rs79164577	Snv	Moderate	**1.49e‐02**	**1.08e‐02**	—	**1.88e‐03**	**1.88e‐03**

(−) no annotation; *P‐value obtained by Fisher's exact test; bold: significant result (*P*‐value ≤ 0.05).

The variants rs4818 and rs6267 present in the *COMT* gene, rs3745274 in the *CYP2B6* gene, and rs149012039 in the *CYP2D6* gene have a high predicted impact, one of them (rs149012039) being an Insertion/Deletion, as well as being statistically significant when compared with all the other five analysis populations. The other high‐impact variant (rs3892097), also in the *CYP2D6* gene, is significant in three populations. The moderate impact variants rs2032582 and rs79164577 show significant differences with four of the five global populations.

## DISCUSSION

MDD is the leading mental disorder worldwide, and prior access to pharmacogenomic tests is of great benefit in accurately indicating the drug and the effective dose for the patient. Thus, the main challenge of pharmacogenomics is to identify genetic variants that may influence different therapeutic responses. However, traditional populations such as the Indigenous people of the Amazon continue to be neglected in this type of research.

The unique genetic profile of the Indigenous population and its high contribution to the Amazonian territory has been the subject of various studies, mainly comparisons with other world populations.^29^, ^30^ It is known that this great differentiation is partly due to the prolonged geographical isolation of this population, as well as endogamous processes, which can influence the fluctuation in the allelic frequency of variants.^12^, ^31^

In addition, most studies focus on European populations, thus emphasizing the importance of pharmacogenomic research in other groups, such as the Indigenous people of the Amazon. The identification of new genetic variants in pharmacogenes that have the potential to be applied as predictive biomarkers of therapeutic success in different classes of drugs can outline specific treatment protocols, with the aim of improving drug efficacy in Indigenous populations, as well as other populations, such as mixed‐race Brazilians. It is important to note that previous studies on Indigenous people have already demonstrated the importance of these new variants as possible biomarkers for defining drug indications, as in Rodrigues (2022), who reported around 167 new variants in pharmacogenes of therapeutic importance. Therefore, this article aimed to understand the genomic profile of the Indigenous population by investigating 9 genes in drug ADME pathways in the treatment of MDD in 12 different Indigenous villages in the Amazon.

Our study identified at least 5 high‐impact genetic variants in the INDG population with differentially significant *P*‐values (*P* < 0.05) in at least three world populations. The rs4818 and rs6267 in the *COMT* gene showed statistical significance in the five world populations when compared to INDG. Previous studies have analyzed rs4818 using a genotyping method in patients with MDD and its association with drug resistance to treatment with antidepressants, finding significant results in the allele frequency of patients carrying the variant in the gene and the pathophysiology of treatment‐resistant depression.^32^ The rs6267, also in the *COMT* gene and identified in the INDG population, is strongly reported for its influence on treatment and an increased risk of developing other psychiatric illnesses, such as schizophrenia.^33^ Catechol‐O‐methyl transferase (*COMT*) is a gene that encodes an enzyme responsible for degrading dopamine, noradrenaline, and adrenaline, acting as a modulator in the prefrontal cortex; additionally, *COMT* and its genetic variants have been studied in different psychiatric disorders associated with altered emotional functions and response to treatments.^34^, ^35^

In addition, three variants with a high predicted impact (rs3745274, rs149012039, and rs3892097) and a significant *P*‐value (*P* < 0.05), one of which is an INDEL, were identified in the INDG population in genes of the CYP family. The cytochrome P450 family or CYP genes is a group of enzymes responsible for the main drug metabolization system, which can be affected by the presence of genetic variants capable of encoding enzymes with normal, reduced, or null activities. In addition, previous studies show the CYP2D6*5 allele as complete deletion of the gene requiring therapeutic adjustments for psychiatric drugs metabolized by CYP.^36^, ^37^

The INDEL variant in the *CYP2D6* gene (rs149012039) has a high frequency in the INDG population (MAF > 10%) and a significant *P*‐value when compared to the five worldwide populations in the 1,000 genomes database. These variants have genetic importance because they alter the amino acid reading matrix and the presence of genetic variants in *CYP2D6* can influence drug metabolization and consequently therapeutic response, where each allele is associated with normal, decreased, absent, or unknown enzyme function, making it one of the most extensively studied genes because it is an important target for implementing pharmacogenomics.^24^, ^38^ These analyses of *CYP2D6* variants in the INDG population not only identified the presence of high‐impact variants such as rs149012039, but also reinforce the role of this population in elucidating alleles of unknown or rare function. The rs202102799 and rs1058172 variants were identified in our study as high‐impact variants in the *CYP2D6* gene. Although haplotyping data for the definition of the CYP2D6*127 allele, which is composed of these two variants, still show an unknown enzymatic function in international databases, the frequency and unique genetic context of this population may be crucial for its functional clarification.^9^, ^23^, ^28^ The prevalence of such variants, when compared to global populations, is a strong indicator of differential pharmacogenomic phenotypes in the Amazonian population.

Another important result of our study was the identification of six new variants, possibly exclusive to the INDG Amazonian population. Among these, a significant number of four variants were identified in CYP family genes, positions 52,919, 52,912, 52,910 in the *CYP2D6* gene and 74,754,842 in the *CYP1A2* gene, as well as a variant in position 114,731,282 of the *HTR2C* gene and an INDEL variant in the 5′ UTR region present in the *HTR2A* gene, position 46,896,758. Genetic variants in untranslated regions (UTRs) have become targets of genomic investigations due to their profound impact on the expression and regulation of initiation in the translation process, implicating them in numerous human diseases.^39^, ^40^

The presence of genetic variants in key genes, such as CYP, discussed earlier, and the 5‐HTR receptor family, can alter the expression and functionality of enzymes and drug transporters involved in ADME pathways, where previous studies have shown a strong connection between the 5‐HTR heterocomplex and the therapeutic response to SSRIs, making it a possible target for the treatment of MDD due to its allosteric receptor–receptor interaction in the plasma membrane in this disease.^41^, ^42^ Recognizing that the 5‐HTR and CYP families are therapeutic targets, we can infer that the new and possibly unique variants found in INDG potentially play an important role in the regulation of these genes and therapeutic efficacy, and further research is needed to understand their true impact.

To demonstrate the genetic uniqueness of the INDG population and its differentiation from other populations around the world, the MDS shows the INDG in a field isolated from the others, with an approximation to the AFR and AMR populations, the latter predicted by their genetic contribution to the historical formation of mixed‐race Americans.

Finally, after analyzing the results, it is possible to reaffirm the need for genomic research studies in Indigenous populations involving clinical molecular biomarkers already described in specialized literature or even undiscovered genetic markers that are specific to Indigenous people. We have limitations such as the small number of INDG individuals who come from isolated and relatively small populations in the Amazon region. Furthermore, the *CYP2C19* gene—gene with an independent and well‐documented role in the metabolism of several antidepressants (e.g., citalopram/escitalopram)—was not included among the nine genes analyzed. The selection was made based on a literature review and consultation of the PharmGKB database with the aim of forming a focused panel of ADME genes relevant to psychotropic drugs that presented evidence in Amazonian Indigenous populations and adequate exomic coverage. This is a preliminary study and did not investigate individuals affected by MDD; however, our results may reveal important information and contribute to the assessment of individual risk for the development of this disease. So far, the knowledge obtained about these populations is very limited to specific genes and does not cover a broader context of the genome involving certain diseases or even therapies aimed at this Indigenous group. Future studies using whole‐genome sequencing (WGS) or targeted pharmacogenomic panels will be essential to achieve a more complete characterization of genetic variability in these populations.

Our data will improve our knowledge of the unique genetic profile of the Indigenous Amazonian population, as well as boosting future clinical research that evaluates the response of this population to conventional treatments for MDD influenced by genetic variants, also helping in studies for highly mixed populations, such as the Brazilian one.

## CONCLUSIONS

We evaluated the presence of genetic variants in nine genes associated with drug ADME pathways for the treatment of MDD in different Indigenous villages in the Brazilian Amazon. Our findings show the presence of well‐elucidated genetic variants and the identification of possible new variants of clinical impact in genes of importance, such as the CYP family and 5‐HTR. This study allows us to reaffirm the unique genetic profile of ancestral populations, such as Indigenous peoples, and of the mixed‐race population of Brazil. Our data may help to identify specific biomarkers that help to understand the individual genetic profiles of Indigenous people and to apply this knowledge to the development of specific treatment protocols guided by pharmacogenomics for these ancestral groups, improving the quality of life of these individuals and the mixed‐race people who descend from them.

## FUNDING

This research was funded by the Conselho Nacional de Desenvolvimento Científico e Tecnológico (CNPq); the Coordenação de Aperfeiçoamento de Pessoal de Nível Superior (CAPES); and the Federal University of Pará (UFPA).

## CONFLICTS OF INTEREST

The authors declared no competing interests for this work.

## AUTHOR CONTRIBUTIONS

K.E.C.A. wrote the manuscript; N.P.C.S., S.E.B.S., J.F.G., and Â.R.‐S. designed the research; K.E.C.A. and M.R.F. performed the research; N.M.S., J.C.G.R., A.M.R.‐S., and S.J.D.S. analyzed the data.

## Supporting information


Table S1.

Table S2.
