# Supplementary material for: Pharmacogenomics of Major Depressive Disorder in Indigenous Amazonian Populations
Source: Clin Pharmacol Ther. 2026 Jan 16;119(5):1331–9. doi: 10.1002/cpt.70207 (PMC13083367; doi:10.1002/cpt.70207)
Supplement: Supplementary file 1 — Table S1. [file CPT-119-1331-s001.docx]

**Supplementary Table 1.** Description of the variants in the *ABCB1, COMT, CYP1A2, CYP2B6, CYP2D6, HTR2A, HTR2C, SLC1A1* and *SLC6A4* genes according to impact modifier and low, in addition to continental populations (African (AFR), American population (AMR), East Asian (EAS), European (EUR), and South Asian (SAS)) described in the 1000 genomes database.

| **Gene** | **Chromossome** | **Position** | **Reference** | **Variant** | **Var Type** | **SNP Id** | **Impact** | **INDG*** | **AFR*** | **AMR*** | **EAS*** | **EUR*** | **SAS*** |
| --- | --- | --- | --- | --- | --- | --- | --- | --- | --- | --- | --- | --- | --- |
| *CYP2D6* | Chr22 | 42142074 | C | T | Snv | rs2267446 | Modifier | 0.0 | 0.108 | 0.146 | 0.542 | 0.202 | 0,157 |
| *CYP2D6* | Chr22 | 42141730 | C | T | Snv | rs149889429 | Modifier | 0.083 | 0.11 | 0.003 | - | - |  |
| *COMT* | Chr22 | 19967393 | AT | A | Indel | rs61143203 | Modifier | 0.0 | 0.154 | 0.278 | 0.423 | 0.167 | 0.178 |
| *ABCB1* | Chr7 | 87509195 | A | G | Snv | rs2235048 | Modifier | 0.666 | 0.850 | 0.563 | 0.601 | 0.481 | 0.421 |
| *ABCB1* | Chr7 | 87563415 | A | G | Snv | rs11975994 | Modifier | 0.052 | 0.141 | 0.402 | 0.628 | 0.416 | 0.598 |
| *CYP2D6* | Chr22 | 42128711 | C | G | Snv | rs112568578 | Modifier | 0.25 | - | - | - | - | - |
| *CYP2D6* | Chr22 | 42143055 | GC | G | Indel | rs368385315 | Modifier | 0.0 | 0.003 | 0.003 | - | - | - |
| *CYP2D6* | Chr22 | 42129714 | G | A | Snv | rs1235509136 | Modifier | 0.015 | - | - | - | - | - |
| *CYP2D6* | Chr22 | 42142061 | G | C | Snv | rs2267444 | Modifier | 0.083 | 0.108 | 0.146 | 0.544 | 0.202 | 0.158 |
| *COMT* | Chr22 | 19964374 | G | C | Snv | rs4646315 | Modifier | 0.421 | 0.124 | 0.222 | 0.123 | 0.187 | 0.249 |
| *SLC1A1* | Chr9 | 4566036 | G | A | Snv | rs73383440 | Modifier | 0.083 | 0.197 | 0.017 | - | 0.001 | - |
| *CYP2D6* | Chr22 | 42142884 | G | T | Snv | rs2743460 | Modifier | 0.15 | 0.101 | 0.163 | 0.015 | 0.224 | 0.152 |
| *SLC1A1* | Chr9 | 4561421 | T | C | Snv | rs72694015 | Modifier | 0.351 | 0.008 | 0.218 | 0.424 | 0.146 | 0.137 |
| *ABCB1* | Chr7 | 87564281 | G | A | Snv | rs4148734 | Modifier | 0.078 | 0.026 | 0.344 | 0.164 | 0.297 | 0.149 |
| *CYP2D6* | Chr22 | 42142988 | G | T | Snv | rs556641825 | Modifier | 0.0 | - | - | 0.001 | 0.001 | - |
| *SLC1A1* | Chr9 | 4561391 | G | C | Snv | rs139557512 | Modifier | 0.083 | - | 0.043 | 0.015 | - | - |
| *CYP2D6* | Chr22 | 42142986 | C | T | Snv | rs371970686 | Modifier | 0.0 | - | - | 0.001 | - | - |
| *COMT* | Chr22 | 19967393 | AT | A | Indel | rs61143203 | Modifier | 0.153 | 0.154 | 0.278 | 0.423 | 0.167 | 0.178 |
| *CYP2B6* | Chr19 | 41006887 | C | T | Snv | rs4803419 | Modifier | 0.0 | 0.082 | 0.352 | 0.436 | 0.320 | 0.342 |
| *CYP1A2* | Chr15 | 74753351 | T | C | Snv | rs4646427 | Modifier | 0.0135 | 0.110 | 0.023 | 0.081 | 0.022 | 0.069 |
| *ABCB1* | Chr7 | 87600185 | C | T | Snv | rs2214102 | Modifier | 1.0 | 0.003 | 0.020 | - | 0.093 | 0.012 |
| *COMT* | Chr22 | 19968739 | G | GC | Indel | rs1463843951 | Modifier | 0.557 | 0.393 | 0.568 | 0.553 | 0.729 | 0.669 |
| *ABCB1* | Chr7 | 87694100 | GT | G | Indel | rs36014243 | Modifier | 0.142 | 0.406 | 0.173 | 0.217 | 0.107 | 0.158 |
| *ABCB1* | Chr7 | 87628672 | C | T | Snv | rs565909048 | Modifier | 0.0 | 0.005 | - | - | - | - |
| *SLC6A4* | Chr17 | 30222791 | C | T | Snv | rs28914827 | Modifier | 0.083 | - | 0.004 | - | 0.017 | 0.014 |
| *ABCB1* | Chr7 | 87570248 | C | A | Snv | rs2235015 | Modifier | 0.031 | 0.392 | 0.151 | 0.071 | 0.207 | 0.181 |
| *HTR2C* | Chrx | 114639531 | G | T | Snv | rs76971695 | Modifier | 0.037 | 0.004 | 0.118 | 0.052 | 0.001 | 0.007 |
| *ABCB1* | Chr7 | 87509216 | A | C | Snv | rs2235047 | Modifier | 0.10 | 0.193 | 0.098 | 0.421 | 0.019 | 0.110 |
| *ABCB1* | Chr7 | 87549827 | A | G | Snv | rs2235033 | Modifier | 0.453 | 0.515 | 0.546 | 0.375 | 0.493 | 0.404 |
| *COMT* | Chr22 | 19967324 | G | A | Snv | rs4646318 | Modifier | 0.031 | 0.069 | 0.062 | 0.064 | 0.051 | 0.079 |
| *ABCB1* | Chr7 | 87549310 | C | T | Snv | rs2235013 | Modifier | 0.453 | 0.515 | 0.546 | 0.375 | 0.493 | 0.404 |
| *SLC1A1* | Chr9 | 4544721 | A | T | Snv | rs45518336 | Modifier | 0.0 | 0.141 | 0.169 | 0.234 | 0.242 | 0.337 |
| *SLC1A1* | Chr9 | 4574045 | C | G | Snv | rs12004839 | Modifier | 0.015 | 0.305 | 0.030 | - | 0.003 | - |
| *SLC1A1* | Chr9 | 4576851 | G | C | Snv | rs301979 | Modifier | 0.781 | 0.244 | 0.220 | 0.278 | 0.290 | 0.393 |
| *CYP2D6* | Chr22 | 42128741 | G | A | Snv | rs113889384 | Modifier | 0.25 | - | - | - | - | - |
| *ABCB1* | Chr7 | 87595730 | G | A | Snv | rs2235074 | Modifier | 0.03 | 0.115 | 0.052 | 0.054 | 0.040 | 0.033 |
| *ABCB1* | Chr7 | 87629088 | G | A | Snv | rs2157928 | Modifier | 0.0 | 0.143 | 0.022 | - | - | - |
| *SLC1A1* | Chr9 | 4561374 | C | T | Snv | rs10974624 | Modifier | 0.075 | 0.106 | 0.295 | 0.466 | 0.279 | 0.191 |
| *SLC6A4* | Chr17 | 30222880 | G | T | Snv | rs6354 | Modifier | 0.918 | 0.328 | 0.140 | 0.119 | 0.213 | 0.158 |
| *SLC1A1* | Chr9 | 4566210 | C | A | Snv | rs7022772 | Modifier | 0.2 | 0.018 | 0.163 | 0.275 | 0.261 | 0.343 |
| *HTR2A* | Chr13 | 46896689 | C | T | Snv | rs6312 | Modifier | 1.0 | 0.826 | 0.944 | 1 | 0.940 | 0.921 |
| *ABCB1* | Chr7 | 87539433 | C | T | Snv | rs4728699 | Modifier | 1.0 | 0.001 | 0.026 | 0.003 | 0.049 | 0.063 |
| *SLC6A4* | Chr17 | 30211514 | C | T | Snv | rs140701 | Modifier | 0.190 | 0.296 | 0.530 | 0.818 | 0.410 | 0.510 |
| *ABCB1* | Chr7 | 87550127 | G | A | Snv | rs2032588 | Modifier | 0.015 | 0.206 | 0.036 | - | 0.070 | - |
| *ABCB1* | Chr7 | 87504154 | T | A | Snv | rs17064 | Modifier | 0.037 | 0.145 | 0.059 | - | 0.073 | 0.001 |
| *CYP2D6* | Chr22 | 42143104 | T | C | Snv | rs71329131 | Modifier | 0.0 | 0.006 | 0.143 | 0.082 | 0.118 | 0.090 |
| *ABCB1* | Chr7 | 87504222 | A | G | Snv | rs28364275 | Modifier | 0.1 | 0.079 | 0.004 | - | 0.001 | - |
| *ABCB1* | Chr7 | 87550882 | C | T | Snv | rs10276036 | Modifier | 0.0 | 0.194 | 0.411 | 0.627 | 0.416 | 0.587 |
| *CYP2B6* | Chr19 | 41004222 | A | T | Snv | rs2279342 | Modifier | 0.283 | 0.044 | 0.170 | 0.185 | 0.118 | 0.165 |
| *SLC1A1* | Chr9 | 4576774 | G | A | Snv | rs1471786 | Modifier | 0.398 | 0.116 | 0.228 | 0.399 | 0.152 | 0.148 |
| *CYP2B6* | Chr19 | 41012868 | C | T | Snv | rs8192719 | Modifier | 0.0 | 0.374 | 0.372 | 0.216 | 0.242 | 0.381 |
| *CYP2D6* | Chr22 | 42128706 | T | C | Snv | rs111564371 | Modifier | 0.25 | - | - | - | - | - |
| *COMT* | Chr22 | 19962712 | C | T | Snv | rs4633 | Low | 0.453 | 0.293 | 0.380 | 0.270 | 0.499 | 0.495 |
| *ABCB1* | Chr7 | 87550285 | A | G | Snv | rs1128503 | Low | 0.370 | 0.136 | 0.403 | 0.627 | 0.416 | 0.587 |
| *HTR2A* | Chr13 | 46895805 | G | A | Snv | rs6313 | Low | 0.101 | 0.393 | 0.354 | 0.588 | 0.436 | 0.422 |
| *HTR2A* | Chr13 | 46892487 | G | A | Snv | rs6305 | Low | 0.083 | 0.002 | 0.026 | - | 0.023 | 0.002 |
| *CYP2D6* | Chr22 | 42141182 | G | A | Snv | rs150262848 | Low | 0.1 | 0.094 | 0.001 | - | - | - |
| *CYP2B6* | Chr19 | 41012413 | C | T | Snv | rs140830969 | Low | 0.083 | - | 0.004 | - | - | - |
| *SLC1A1* | Chr9 | 4574022 | A | C | Snv | rs12682807 | Low | 0.085 | 0.005 | 0.102 | 0.255 | 0.119 | 0.080 |
| *CYP2D6* | Chr22 | 42128844 | G | A | Snv | rs1379103867 | Low | 0.083 | - | - | - | - | - |
| *CYP2B6* | Chr19 | 41012868 | C | T | Snv | rs8192719 | Low | 0.0 | 0.374 | 0.372 | 0.216 | 0.242 | 0.381 |
| *CYP2D6* | Chr22 | 42127537 | A | G | Snv | rs28371726 | Low | 0.083 | - | - | - | - | - |
| *CYP2D6* | Chr22 | 42128922 | A | G | Snv | rs111606937 | Low | 0.25 | - | - | - | - | - |
| *CYP2D6* | Chr22 | 42142508 | G | A | Snv | rs56023519 | Low | 0.166 | 0.489 | 0.231 | 0.569 | 0.295 | 0.278 |
| *SLC1A1* | Chr9 | 4583074 | C | A | Snv | rs568302878 | Low | 0.083 | 0.001 | - | - | - | - |
| *COMT* | Chr22 | 19963684 | C | G | Snv | rs4818 | Low | 0.023 | 0.170 | 0.295 | 0.341 | 0.403 | 0.315 |
| *CYP1A2* | Chr15 | 74753351 | T | C | Snv | rs4646427 | Low | 0.013 | 0.110 | 0.023 | 0.081 | 0.022 | 0.069 |
| *CYP2D6* | Chr22 | 42141242 | G | A | Snv | rs2743457 | Low | 0.083 | 0.299 | 0.157 | 0.564 | 0.203 | 0.163 |
| *SLC1A1* | Chr9 | 4564432 | G | A | Snv | rs2228622 | Low | 0.453 | 0.192 | 0.458 | 0.236 | 0.410 | 0.431 |
| *ABCB1* | Chr7 | 87509329 | A | G | Snv | rs1045642 | Low | 0.398 | 0.150 | 0.428 | 0.398 | 0.518 | 0.575 |
| *CYP2D6* | Chr22 | 42128793 | A | G | Snv | rs28371713 | Low | 0.25 | - | - | - | - | - |
| *SLC6A4* | Chr17 | 30212795 | G | A | Snv | rs55908624 | Low | 0.0 | - | 0.017 | 0.001 | - | 0.002 |
| *COMT* | Chr22 | 19962745 | G | A | Snv | rs740602 | Low | 0.008 | - | - | - | - | - |
| *CYP2D6* | Chr22 | 42129754 | G | A | Snv | rs1081003 | Low | 0.078 | 0.092 | 0.050 | 0.570 | 0.024 | 0.082 |
| *SLC1A1* | Chr9 | 4576680 | T | C | Snv | rs301430 | Low | 0.460 | 0.487 | 0.617 | 0.412 | 0.672 | 0.562 |

(-) No annotation; (*) Minor allele frequencies; INDG: Indigenous population, AFR: African population, AMR: American population, EAS: East Asian population, EUR: European population, SAS: South Asian population.
