# Supplementary material for: Pharmacogenomics of Major Depressive Disorder in Indigenous Amazonian Populations
Source: Clin Pharmacol Ther. 2026 Jan 16;119(5):1331–9. doi: 10.1002/cpt.70207 (PMC13083367; doi:10.1002/cpt.70207)
Supplement: Supplementary file 2 — Table S2. [file CPT-119-1331-s002.docx]

**Supplementary Table 2.** Paired comparison (*p*-value) with significant results of allele frequencies in the Indigenous population (INDG) and continental populations (African (AFR), American (AMR), East Asian (EAS), European (EUR) and South Asian (SAS)) described in the1000 genomes database.

| **Gene** | **SNP Id** | **Var Type** | **Impact** | **INDG vs AFR*** | **INDG vs AMR*** | **INDG vs EAS*** | **INDG vs EUR*** | **INDG vs SAS*** |
| --- | --- | --- | --- | --- | --- | --- | --- | --- |
| *ABCB1* | rs10276036 | Snv | Modifier | **7.53e-02** | **3.32e-12** | **3.46e-25** | **3.32e-12** | **1.72e-22** |
| *ABCB1* | rs11975994 | Snv | Modifier | - | **1.14e-04** | **1.67e-16** | **2.42e-05** | **4.69e-15** |
| *ABCB1* | rs2032582 | Snv | Moderate | - | **5.63e-04** | **2.42e-05** | **1.08e-05** | **1.80e-15** |
| *ABCB1* | rs2214102 | Snv | Modifier | **3.91e-68** | **7.15e-63** | - | **9.49e-52** | **2.46e-64** |
| *ABCB1* | rs2235015 | Snv | Modifier | **1.52e-06** | **3.77e-03** | - | **7.14e-04** | **4.31e-05** |
| *ABCB1* | rs2235048 | Snv | Modifier | **1.88e-03** | - | - | **1.09e-02** | **4.59e-04** |
| *ABCB1* | rs4728699 | Snv | Modifier | **3.90e-74** | **7.15e-69** | **3.90e-74** | **1.20e-64** | **3.44e-62** |
| *COMT* | rs4646315 | Snv | Modifier | **5.24e-07** | **1.88e-03** | **5.24e-07** | **2.23e-04** | **1.08e-02** |
| *CYP2B6* | rs4803419 | Snv | Modifier | **3.76e-03** | **4.77e-15** | **1.86e-19** | **1.42e-13** | **1.05e-14** |
| *CYP2D6* | rs113472173 | Snv | Moderate | **1.49e-04** | **1.09e-02** | - | **1.88e-02** | - |
| *CYP2D6* | rs28371706 | Snv | Moderate | **1.08e-02** | **1.09e-02** | - | **1.88e-03** | - |
| *CYP2D6* | rs3021082 | Snv | Moderate | **1.33e-07** | - | **9.17e-11** | **3.02e-02** | - |
| *CYP2D6* | rs71329131 | Snv | Modifier | - | **1.47e-05** | **3.76e-03** | **1.25e-04** | **1.03e-03** |
| *CYP2D6* | rs79164577 | Snv | Moderate | **1.49e-02** | **1.08e-02** | - | **1.88e-03** | **1.88e-03** |
| *HTR2A* | rs6312 | Snv | Modifier | **7.32e-07** | **2.65e-02** | - | **1.36e-02** | **3.76e-03** |
| *SLC1A1* | rs10974624 | Snv | Modifier | - | **3.44e-04** | **6.90e-18** | **1.06e-04** | **2.84e-02** |
| *SLC1A1* | rs1471786 | Snv | Modifier | **1.38e-06** | **9.89e-03** | - | **3.69e-05** | **3.69e-05** |
| *SLC1A1* | rs301979 | Snv | Modifier | **3.64e-11** | **1.06e-12** | **6,09e-09** | **1.62e-08** | **1.46e-03** |
| *SLC1A1* | rs45518336 | Snv | Modifier | **1.47e-05** | **7.32e-07** | **1.43e-09** | **6.52e-10** | **2.47e-14** |
| *SLC1A1* | rs73383440 | Snv | Modifier | **3.25e-02** | **3.27e-02** | - | **1.88e-03** | - |
| *SLC6A4* | rs140701 | Snv | Modifier | - | **6.27e-02** | **6.46e-18** | **5.66e+02** | **4.60e-01** |
| *SLC6A4* | rs28914827 | Snv | Modifier | - | **1.09e-02** | - | **3.27e-02** | **3.27e-02** |
| *SLC6A4* | rs6354 | Snv | Modifier | **5.80e-23** | **1.52e-38** | **4.31e-41** | **7.49e-32** | **5.86e-37** |
| *SLC6A4* | rs6355 | Snv | Modifier | **1.08e-02** | - | - | **3.27e-02** | **3.27e-02** |

(-) No annotation; *p-value obtained by Fisher’s exact test; bold: significant result (p-value ≤ 0.05).
